# Diffusion kurtosis MRI as a predictive biomarker of response to neoadjuvant chemotherapy in high grade serous ovarian cancer

**DOI:** 10.1038/s41598-019-47195-4

**Published:** 2019-07-24

**Authors:** Surrin S. Deen, Andrew N. Priest, Mary A. McLean, Andrew B. Gill, Cara Brodie, Robin Crawford, John Latimer, Peter Baldwin, Helena M. Earl, Christine Parkinson, Sarah Smith, Charlotte Hodgkin, Ilse Patterson, Helen Addley, Susan Freeman, Penny Moyle, Mercedes Jimenez-Linan, Martin J. Graves, Evis Sala, James D. Brenton, Ferdia A. Gallagher

**Affiliations:** 1 Department of Radiology, Box 218, University of Cambridge, Cambridge, CB2 0QQ United Kingdom; 2 Cambridge University Hospitals NHS Foundation Trust, Addenbrooke’s Hospital, Cambridge, CB2 0QQ United Kingdom; 3 Cancer Research UK Cambridge Institute, University of Cambridge, Cambridge, CB2 0RE United Kingdom

**Keywords:** Cancer imaging, Chemotherapy

## Abstract

This study assessed the feasibility of using diffusion kurtosis imaging (DKI) as a measure of tissue heterogeneity and proliferation to predict the response of high grade serous ovarian cancer (HGSOC) to neoadjuvant chemotherapy (NACT). Seventeen patients with HGSOC were imaged at 3 T and had biopsy samples taken prior to any treatment. The patients were divided into two groups: responders and non-responders based on Response Evaluation Criteria In Solid Tumours (RECIST) criteria. The following imaging metrics were calculated: apparent diffusion coefficient (ADC), apparent diffusion (D_app_) and apparent kurtosis (K_app_). Tumour cellularity and proliferation were quantified using histology and Ki-67 immunohistochemistry. Mean K_app_ before therapy was higher in responders compared to non-responders: 0.69 ± 0.13 versus 0.51 ± 0.11 respectively, *P* = 0.02. Tumour cellularity correlated positively with K_app_ (rho = 0.50, *P* = 0.04) and negatively with both ADC (rho = −0.72, *P* = 0.001) and D_app_ (rho = −0.80, *P* < 0.001). Ki-67 expression correlated with K_app_ (rho = 0.53, *P* = 0.03) but not with ADC or D_app_. In conclusion, K_app_ was found to be a potential predictive biomarker of NACT response in HGSOC, which suggests that DKI is a promising clinical tool for use oncology and radiology that should be evaluated further in future larger studies.

## Introduction

Ovarian cancer has the highest mortality of any gynaecological malignancy in the developed world. Disease prognosis depends on tumour subtype and the stage at diagnosis with high grade serous ovarian cancer (HGSOC) accounting for the majority of deaths^1^. The best treatment for HGSOC is with a combination of chemotherapy and cytoreductive surgery^2^.

The first line chemotherapy choice for HGSOC is a platinum-based drug together with a taxane^3^, both of which inhibit cell division. This chemotherapy treatment combination is associated with significant morbidity due to medication side effects, and has a complete remission rate of only around 50%^3,4^. Newer targeted therapies based around DNA damage repair inhibition^5,6^, vascular growth factor inhibition^6^ and immune checkpoint inhibition^7^ are now being developed that may provide alternative treatment options to HGSOC patients in the future. With the availability of new therapies, there is an increasing need for methods to both predict and detect the response to treatment in HGSOC at the earliest timepoints possible, so that the best personalized therapies can be selected for individual patients.

Diffusion weighted imaging (DWI) has previously been shown to identify early treatment response in HGSOC by reporting on the cytotoxic effect of platinum-based chemotherapy^8^. In this study an extended version of diffusion MRI modelling, diffusion kurtosis imaging (DKI), is evaluated as a predictive biomarker of neoadjuvant chemotherapy (NACT) response before the initiation of treatment.

Conventional clinical DWI assumes that the diffusion of water follows a Gaussian distribution. This approach however over simplifies the movement of water in tissue, as the heterogenous spatial distribution of microstructures that obstruct diffusion (such as the membranes of cells and organelles) imparts a positive peak to the Gaussian model, termed kurtosis. Kurtosis is more apparent at higher diffusion weightings and DKI modelling is relatively easy to translate into clinical practice through the use of appropriate b-values^9^.

DKI has been shown to measure tissue heterogeneity and to correlate with expression of the proliferation marker Ki-67 in several malignancies, including ovarian cancer^10–12^. Given that Ki-67 is known to identify cancers that are sensitive to chemotherapeutic agents that target proliferating cells^13,14^, in this study we hypothesized that DKI may be able to predict the response of HGSOC to chemotherapy drugs that inhibit cell division like carboplatin and paclitaxel. There is already some evidence to support this property of DKI in nasopharyngeal cancer^15^ and in this work we present the first exploratory study of the ability of DKI to predict the response of HGSOC in patients undergoing standard of care NACT before the start of treatment.

## Materials and Methods

### Study conduct

This was a single centre prospective, observational study on a consecutive sample of seventeen treatment-naïve patients with new diagnoses of HGSOC. Included participants had no previous cancer treatment or surgery and no contraindications to MRI. The recruitment was part of the MISSION-ovary (Molecular Imaging and Spectroscopy with Stable Isotopes in Oncology and Neurology) research study for investigating the use of novel MRI methods in ovarian cancer: ClinicalTrials.gov Identifier, NCT03526809. Institutional review board approval was obtained for all study related procedures (South Cambridge Research Ethics Committee reference 15/EE/0378) and written informed consent was obtained from all participants. All study related procedures were carried out in accordance with the research ethics guidelines outlined in the Declaration of Helsinki. The research MRI did not change clinical management, which was based on standard of care computed tomography (CT).

### MRI technique and image analysis

A 3 T MRI scanner (Discovery MR750, GE Healthcare, Waukesha WI) and a 32-channel cardiac array coil were used to perform DWI and T_2_-weighted imaging in participants between one and seven days before the start of chemotherapy treatment. 20 mg of intravenous hyoscine butylbromide was given 5 min prior to imaging to reduce artefacts from bowel motion. Full scan parameters are listed in Table 1.

**Table 1:** Table of imaging parameters. T_2_-weighted and diffusion imaging parameters.

Imaging parameter	T2-weighted	Diffusion weighted imaging
TR	4000 ms	6000 ms
TE	91.1 ms	94 ms
flip angle	90°	90°
slice thickness	6 mm	6 mm
FoV	34.0 cm × 29.9 cm	34.0 cm × 29.9 cm
matrix	256 × 256	128 × 112
signal averages	8	4
parallel imaging	—	ASSET, factor 2
bandwidth	99.8 kHz	±142 kHz
total scan time	1 min 54 sec	7 min 42 s
b-values	—	100, 500, 900, 1300 and 1700 s/mm^2^

TR = repetition time, TE = echo time, FoV = field of view.

Apparent diffusion (D_app_, in mm^2^/s) and apparent kurtosis (K_app_, unitless) were calculated with in-house software written in MATLAB R2018a (The MathWorks Inc., Natick, MA), by performing a pixel-wise non-linear fit to the bi-exponential diffusion kurtosis model described in equation 1^16^.

1 $${\rm{S}}({\rm{b}})={{\rm{S}}}_{0}\,.\,{{\rm{e}}}^{(-{\rm{b}}.{{\rm{D}}}_{{\rm{app}}}+\frac{1}{6}.{{\rm{b}}}^{2}.{{{\rm{D}}}_{{\rm{app}}}}^{2}.{{\rm{K}}}_{{\rm{app}}})}$$

where S(b) is signal intensity at each b-value, and S_0_ is signal intensity with no diffusion weighting. Apparent diffusion coefficient (ADC) values, in mm^2^/s were also calculated using conventional mono-exponential Gaussian diffusion modelling from the images with b-values of 100, 500 and 900 s/mm^2^. Regions of interest (ROIs) were drawn on the D_app_ maps to reduce errors due to image distortion known to occur between T_2_-weighted and diffusion images^17^. ROIs were drawn with OsiriX (version 3.8.1, Pixmeo, Geneva, Switzerland) by a radiologist, with 8 years of attending experience in oncological imaging and who was kept blind to treatment response and tissue analysis results. The ROIs were placed around all solid cancerous lesions, with care taken to exclude cystic and necrotic regions and imported onto the ADC and K_app_ maps, which were assumed to be co-registered as they were derived from the same set of DWI images. For each patient all tumour ROIs present in the abdomen and pelvis were combined into a volume of interest (VOI) to derive single ADC, D_app_ and K_app_ values from each patient for analysis. Intraobserver and interobserver variability were assessed by ROIs drawn by a second observer with four years of experience as a general medical doctor and three year of specialist experience as a radiology researcher in oncological imaging and diffusion MRI.

### Response evaluation

Response to NACT was assessed according to Response Evaluation Criteria In Solid Tumours (RECIST) criteria version 1.1^18^, using contrast enhanced CT scans performed as part of the patients’ regular clinical management. These were a baseline CT scan before the initiation of chemotherapy and a second CT scan up to one week after the third cycle of chemotherapy. Response was evaluated at the gynaecologic oncology multi-disciplinary team (MDT) meeting by a consensus decision from gynaecologic radiologists, oncologists, surgeons and histopathologists after review of the CT scans. All MDT members were kept blind to the research MRI and tissue analysis results. Participants with 30% or greater reduction in disease, i.e. a RECIST Complete Response (CR) or Partial Response (PR) were classified as responders and those with Stable Disease (SD) or Progressive Disease (PD) were classified as non-responders.

### Tissue processing and immunohistochemistry

Tumour samples were collected from either ovarian or peritoneal cancer deposits before treatment either by ultrasound-guided needle biopsy or a surgical procedure in the cases of lesions that were not accessible through the percutaneous route. Tissue was fixed in formalin and embedded into paraffin blocks. 3 µm sections were cut from the blocks and stained with H&E (haematoxylin and eosin) and Ki-67 (Dako Cat# M7240). Staining was carried out using Leica’s Polymer Refine Detection System (DS9800) automated Bond platform. This platform included a post primary of rabbit anti-mouse IgG (<10 µg/mL) in 10% (v/v) animal serum plus tris-buffered saline/0.09% (ProClin™ 950) and a polymer of anti-rabbit poly-HRP-IgG (<25 µg/mL) in 10% (v/v) animal serum plus tris-buffered saline/0.09% (ProClin™ 950). Bright-field scanning was performed on an Aperio AT2 scanner (Leica) to digitize slides for automated analysis. Quantification of Ki-67 staining and of the number of cells per unit area, as an estimate of cellularity (cells/µm^2^), were performed using the multiplex IHC V1.2 module of Halo histology image analysis software (Indica labs v2.1.1637.11). Cells with Ki-67 staining greater than an optical density of 0.31 were considered positive. The operator of the analytic software was blinded to MRI and treatment response results.

### Statistical methods

Statistical analysis was performed in R (version 2.15.3, R Foundation for Statistical Computing, Vienna, Austria) and a *P* value of 0.05 was used as the cut-off to indicate significance. Intraobserver and interobserver agreement were assessed using the intraclass correlation coefficient (ICC). When testing for differences in means between groups, the Shapiro–Wilk test was used to assess for normality of data. Student’s t-test or the Mann-Whitney *U* test was then applied for evaluations on normally and non-normally distributed data respectively. Immunohistochemistry and histology results were compared to the diffusion imaging metrics using Spearman’s correlation.

## Results

### Study population

Seventeen patients were recruited to this study. Mean age was 66.6 ± 9.4 (mean ± S.D.) years and age range was 43 to 81 years old. Population demographics are summarized in Table 2. After MRI imaging, 15 of the 17 participants went on to have NACT treatment with a combination of carboplatin and paclitaxel. The remaining 2 patients (one of whom had Stage 1 cancer) were treated by the decisions of their clinical teams with primary surgery and adjuvant chemotherapy and therefore could not be investigated for NACT treatment response as part of this study.

**Table 2:** Characteristics of study population. Population demographics of patients recruited.

Feature	Value
**Number of patients**	17
**Age at diagnosis, mean (range) (years)**	66.6 (43 to 81)
**ECOG performance status (number of patients)**	
0–2	13
3–4	4
**FIGO stage (number of patients)**	
I	0
II	1*
III	12
IV	4
**Serum CA 125 at diagnosis (IU/ml) (number of patients)**	
0–100	4
100–500	5
>500	8
**Volume of ROIs analysed (number of patients)**	
0 to 25 ml	0
>25 to 50 ml	3
>50 to 100 ml	8
>100 ml	6
**Treatment pathway**	
Neoadjuvant treatment	15
Adjuvant treatment	2
**RECIST response on CT**	
Complete response (CR)	0
Partial response (PR)	5
Stable disease (SD)	8
Progressive disease (PD)	2

ECOG = Eastern Cooperative Oncology Group, FIGO = Fédération Internationale de Gynécologie et d’Obstétrique, ROI = region of interest, RECIST = Response Evaluation Criteria In Solid Tumours, CA 125 = cancer antigen 125, NACT = neoadjuvant chemotherapy, S.D. = standard deviation. *The one FIGO stage II patient in this cohort underwent treatment with primary surgery followed by adjuvant chemotherapy.

### Imaging

**Figure 1:**
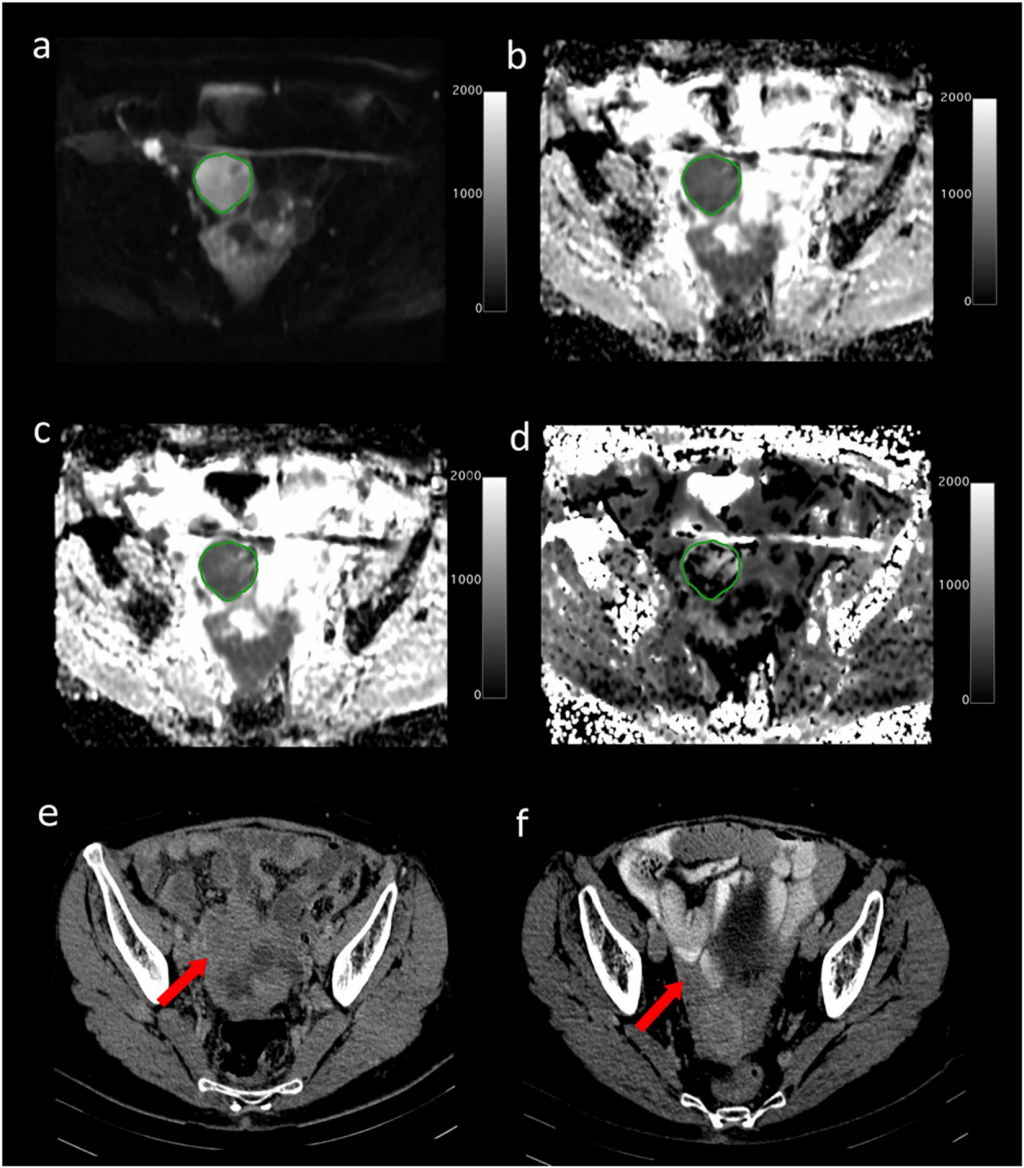
Axial MRI images from a 63-year old high grade serous ovarian cancer patient who had a good response to neo-adjuvant chemotherapy. (**a**) DWI at b = 1300 s/mm^2^. Scale bar represents signal intensity; (**b**) ADC map with tumour ROI shown. Scale bar represents ADC in mm^2^/s × 1000; (**c**) D_app_ map. Scale bar represents D_app_ in mm^2^/s × 1000; (**d**) K_app_ map. Scale bar represents K_app_ × 1000; Axial CT scans following intravenous contrast medium: (**e**) before treatment; (**f**) after treatment, depicting a RECIST Partial Response (PR).

There was a good fit of DWI images to the DKI model for the VOIs analysed. Figure 1 shows an example of a typical DWI image and the diffusion parameter maps for a 63-year old HGSOC patient who responded well to NACT. The CT scans for this patient before and after therapy are also shown.

### Intraobserver and interobserver variability

There was good intraobserver and interobserver agreement for all diffusion metrics measured. Results are summarized in Table 3.

**Table 3:** Intraobserver and interobserver variability for diffusion imaging metrics.

Diffusion metric	Intraobserver ICC	Interobserver ICC
ADC	0.971 (0.967 to 0.972)	0.977 (0.975 to 0.978)
D_app_	0.968 (0.965 to 0.971)	0.974 (0.971 to 0.976)
K_app_	0.989 (0.986 to 0.981)	0.989 (0.986 to 0.982)

ICC = intraclass coefficient correlation, ADC = apparent diffusion coefficient, D_app_ = apparent diffusion, K_app_ = apparent kurtosis. Values in brackets represent the 95% confidence interval.

### Predicting treatment response

**Figure 2:**
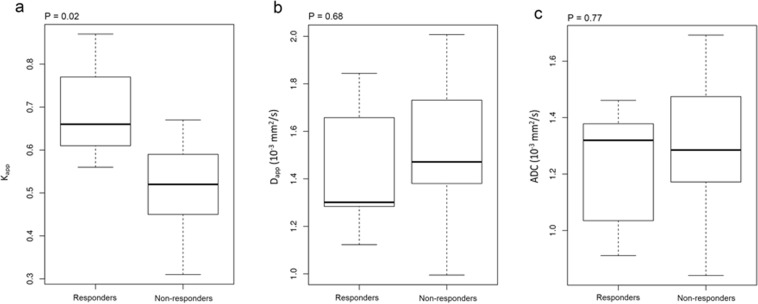
Box-and-whisker plots showing median and inter-quartile ranges of diffusion parameters for responders and non- responders to neoadjuvant chemotherapy. (**a**) K_app_; (**b**) D_app_; (**c**) ADC.

Of the 15 patients to undergo NACT, there were five RECIST responders and ten non-responders. A significant difference was found in the pre-treatment mean K_app_ between the responders and non-responders: 0.69 ± 0.13 versus 0.51 ± 0.11 (mean ± S.D.) respectively; Mann-Whitney *U* test, *P* = 0.02 for a difference between these two groups. D_app_ was not found to be significantly different between responders and non-responders: 1.44 ± 0.30 × 10^−3^ mm^2^/s versus 1.51 ± 0.32 × 10^−3^ mm^2^/s respectively, *P* = 0.68. The difference in ADC between responders and non-responders was similarly non-significant: 1.22 ± 0.24 × 10^−3^ mm^2^/s versus 1.30 ± 0.27 × 10^−3^ mm^2^/s respectively, *P* = 0.77. Boxplots of the median K_app_, D_app_ and ADC values for the responder and non-responder groups are shown in Fig. 2.

### Correlation with cellularity and Ki-67 expression

Localization of the Ki-67 stain was to the nucleus of cells in all cases as expected. Ki-67 staining was also subjectively observed to be greater in tissue that was confirmed as cancerous on H&E, which is consistent with the expression pattern of this protein that is known to be upregulated in ovarian cancer^19^.

**Figure 3:**
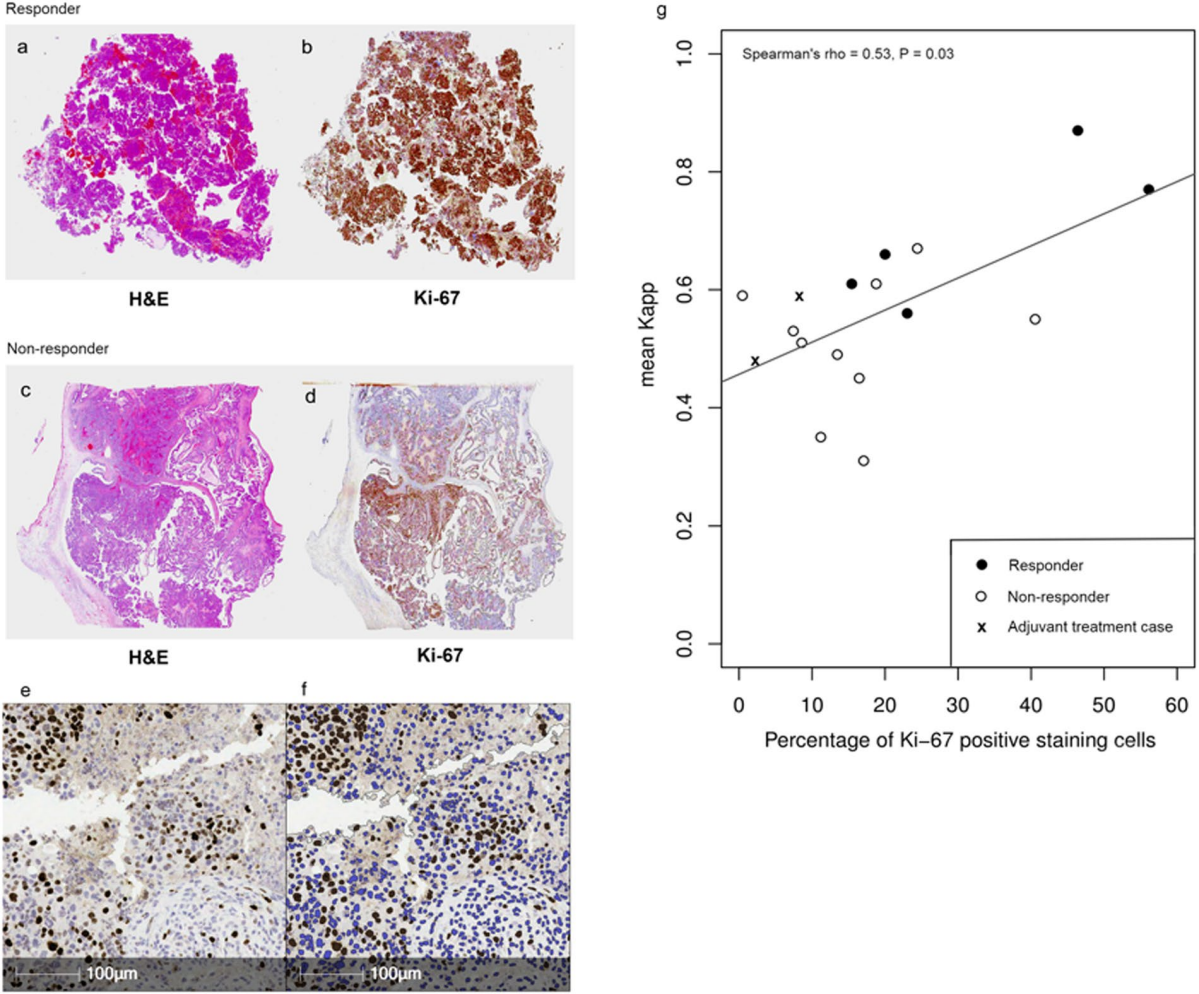
Examples of histology from a responder and a non-responder. (**a**) 1x magnification H&E slide of responder; (**b**) 1x magnification Ki-67 staining from responder (positive tissue shown in brown and negative tissue shown in blue); (**c**) 1x magnification H&E slide of non-responder; (**d**) 1x magnification Ki-67 staining from non-responder (positive tissue shown in brown and negative tissue shown in blue); (**e**) 20x magnification of Ki-67 staining in a HGSOC patient, with positive cells in dark brown and background counter staining in blue; (**f**) automated image segmentation in Halo for quantification of Ki-67 staining. Positive cells are shown in dark brown and negative cells are shown in blue. (**g**) Scatterplot of mean tissue K_app_ against percentage of cells positive for Ki-67 staining (optical density > 31). White circles indicate responders, black circles indicate non-responders and crosses indicate the two patients treated with primary surgery before starting adjuvant chemotherapy.

Figure 3 shows the appearances of the H&E and Ki-67 staining for a responder (Fig. 3a,b) and a non-responder (Fig. 3c,d) to NACT. An example of the automated segmentation of Ki-67 positive cells in Halo is shown in Fig. 3e,f, which illustrates the accuracy of cell classification by the software.

Cellularity exhibited a positive correlation with K_app_ (Spearman rho = 0.49, *P* = 0.04) and negative correlations with both ADC (rho = −0.77, *P* = 0.02) and D_app_ (rho = −0.73, *P* = 0.03). K_app_ correlated positively with the percentage of cells expressing Ki-67 (rho = 0.53, *P* = 0.03), but ADC and D_app_ did not correlate with Ki-67 (*P* = 0.55 and *P* = 0.15 respectively). A scatterplot of mean tumour K_app_ against Ki-67 quantification is shown in Fig. 3g, also identified on this plot are the responder and non-responder NACT cases and the two adjuvant treatment cases.

## Discussion

This study demonstrated that measurements of the non-Gaussian movement of water with DKI may predict the response to neoadjuvant chemotherapy in HGSOC patients. Tumours with a higher mean K_app_ before the start of chemotherapy were found to respond better to treatment whereas neither conventional ADC, nor its equivalent calculated from the DKI acquisition (D_app_), could effectively differentiate responders from non-responders. All three diffusional metrics correlated with cellularity, which was expected as cells form the major barrier to the diffusion of water in tissue^20–22^. Histopathology results also confirmed that the previously reported relationships between diffusion and cellularity^20,23^ and between K_app_ and Ki-67^10,11^ demonstrated in other cancers are also present in this HGSOC patient cohort.

Conventional DWI assumes that the movement of water in tissue is Gaussian. This assumption is problematic in malignancy however, as the structural complexity and heterogeneity within tumours can produce non-Gaussian patterns of diffusion. DKI attempts to address this complication through the inclusion of an additional parameter in the diffusion model, K_app_, that quantifies the kurtosis aspect of the deviation of the imaging signal from a purely mono-exponential Gaussian distribution.

As the magnitude of the K_app_ term in DKI relates to tissue heterogeneity^16^ and heterogeneity in turn is used to help determine tumour grade on histopathology, in some malignancies DKI has been studied for its diagnostic value in tumour grading. Previous research has already demonstrated that DKI can differentiate grade II and III gliomas^12,24^, low grade and high grade prostate cancer^25,26^ and borderline from malignant epithelial ovarian tumours^10^. In the case of epithelial ovarian cancer however, for the one DKI study that was previously performed, K_app_ was not shown to be superior to conventional ADC measurements at diagnosing grade^10^. Additionally, for HGSOC, which is the most clinically relevant subtype of epithelial ovarian cancer, due to its frequency and high mortality, there is no widely accepted subdivision of tumour grading, against which DKI could be easily assessed, as moderately differentiated serous ovarian cancer is no longer believed to be a valid subclassification of the disease^27,28^.

The treatment response findings presented here may be explained by the higher cellular density and microstructural heterogeneity that is present in rapidly proliferating tissue, which can be probed histologically with Ki-67 and non-invasively by K_app_. Rapidly dividing and heterogeneous tumours may be more sensitive to therapies that target cellular replication, such as carboplatin which inhibits DNA synthesis required for new cell development^29^ and paclitaxel which disrupts the microtubule formation necessary for mitosis^30^. Further to this, more proliferative ovarian cancer subtypes like HGSOC are known to respond better to chemotherapy than low grade serous ovarian cancer^31^. The low proliferation rates in epithelial ovarian cancer have been shown previously to relate to chemoresistance^32^ and a number of other high Ki-67 expressing cancers are sensitive to chemotherapy^13,14^. These previous studies all provide evidence to support a true relationship between cellular proliferation in HGSOC and a response to NACT treatment.

Besides the prediction of response to NACT, DKI in HGSOC could also find a clinical role in investigating tumour microstructure and growth in conjunction with other immunohistochemistry and histological markers. Unlike histopathological measurements that are taken from small biopsy samples of tumour tissue that have undergone changes during traumatic sampling and fixation, DKI can non-invasively probe cellularity and proliferation in entire tumour volumes *in vivo*. HGSOC is known to be heterogeneous^33,34^ and a biopsy sample may not always be representative of the Ki-67 expression and cellularity across the complete tumour. DKI measurements which are performed by imaging the whole tumour volume may therefore provide more complete information on tumour biology that may be complementary to that gained from biopsy specimens alone. DKI metrics assess the heterogeneity of water movement and how this differs from a normal Gaussian distribution within an individual voxel. In this study the patient number was too small to permit reliable hypothesis testing of the multiple parameters that would be produced by histogram or textural analysis. Future work with a larger number of patients could however extend this study to assess intervoxel heterogeneity of water movement by using histogram analysis^35,36^ or Haralick textural features^37^.

The conclusions that can be drawn from this study are limited by the small sample size, which restricts the scope for wider interpretation of the results; however, given that imaging findings correlated with the histological analysis and provided a mechanistic explanation for the results observed, there is strong support that the findings here are based on a real biological difference between groups which can be detected on imaging, rather than a statistical aberration. The tissue used to quantify Ki-67 expression and cellularity was also subject to sampling error as histological specimens from a small tissue sample were compared to the imaging results derived from the whole tumour burden of patients. This type of sampling error is unfortunately unavoidable when biopsies from large, heterogenous tumours must be compared to imaging findings; despite this limitation, the correlation between imaging and histology is once again grounded in a biological rationale for the imaging results and reemphasizes the potential clinical utility of combining the detailed histological data acquired from a small biopsy sample with the multiparametric imaging data acquired at lower resolution but from a larger volume of tumour. Other factors that may have influenced treatment response but were not considered here include: the initial tumour burden of patients, the stage of the disease at recruitment, patient co-morbidities and genetic factors such as the presence of *BRCA* and *TP53* mutations that can impact on the effectiveness of chemotherapy^38–40^.

In summary, the results of this study suggest that in HGSOC there may be a clinically relevant relationship between DKI-derived diffusion metrics and the response of the cancer to neo-adjuvant chemotherapy, particularly involving drugs that target cell proliferation. These findings have the potential to be applied to stratify treatment options in ovarian cancer and to rapidly escalate patients to alternative targeted or combinational therapeutic approaches, while reducing morbidity from the side effects of less efficacious drugs. It is also possible that DKI may offer clinical value as an adjunct to histopathology for the measurement of ovarian cancer proliferation and cellularity as it derives from a larger tissue volume. This study therefore provides preliminary data for larger trials to confirm these results and to further explore the applications of DKI in HGSOC patients.
